# Toughness Condition for a Graph to Be a Fractional *(g*, *f*, *n)*-Critical Deleted Graph

**DOI:** 10.1155/2014/369798

**Published:** 2014-07-09

**Authors:** Wei Gao, Yun Gao

**Affiliations:** ^1^School of Information Science and Technology, Yunnan Normal University, Kunming 650500, China; ^2^Editorial Department of Yunnan Normal University, Kunming 650092, China

## Abstract

A graph *G* is called a fractional (*g*, *f*)-deleted graph if *G* − {*e*} admits a fractional (*g*, *f*)-factor for any *e* ∈ *E*(*G*). A graph *G* is called a fractional (*g*, *f*, *n*)-critical deleted graph if, after deleting any *n* vertices from *G*, the resulting graph is still a fractional (*g*, *f*)-deleted graph. The toughness, as the parameter for measuring the vulnerability of communication networks, has received significant attention in computer science. In this paper, we present the relationship between toughness and fractional (*g*, *f*, *n*)-critical deleted graphs. It is determined that *G* is fractional (*g*, *f*, *n*)-critical deleted if *t*(*G*) ≥ (*(b* ^2^ − 1 + *bn*)/*a)*.

## 1. Introduction

All graphs considered in this paper are finite, are loopless, and are without multiple edges. The notation and terminology used but undefined in this paper can be found in [1]. Let *G* be a graph with the vertex set *V*(*G*) and the edge set *E*(*G*). For a vertex *x* ∈ *V*(*G*), we use *d*_*G*_(*x*) and *N*_*G*_(*x*) to denote the degree and the neighborhood of *x* in *G*, respectively. Let *δ*(*G*) denote the minimum degree of *G*. For any *S*⊆*V*(*G*), the subgraph of *G* induced by *S* is denoted by *G*[*S*].

The problem of fractional factor can be considered as a relaxation of the well-known cardinality matching problem. It has wide-ranging applications in areas such as scheduling, network design, and the combinatorial polyhedron. For instance, several large data packets are to be sent to various destinations through several channels in a communication network. The efficiency of this work can be improved if large data packets are to be partitioned into small parcels. The feasible assignment of data packets can be seen as a fractional flow problem and it becomes a fractional factor problem when the destinations and sources of a network are disjoint.

Suppose that *g* and *f* are two integer-valued functions on *V*(*G*) such that 0 ≤ *g*(*x*) ≤ *f*(*x*) for all *x* ∈ *V*(*G*). A* fractional *(*g*, *f*)*-factor* is a function *h* that assigns to each edge of a graph *G* a number in [0,1] so that for each vertex *x* we have *g*(*x*) ≤ ∑_*e*∈*E*(*x*)_ *h*(*e*) ≤ *f*(*x*). If *g*(*x*) = *a* and *f*(*x*) = *b* for all *x* ∈ *V*(*G*), then a fractional (*g*, *f*)-factor is a fractional [*a*, *b*]-factor. Moreover, if *g*(*x*) = *f*(*x*) = *k* for all *x* ∈ *V*(*G*), then a fractional (*g*, *f*)-factor is just a fractional *k*-factor. Throughout this paper, *k* ≥ 1 is an integer, and we will not reiterate it again.

A graph *G* is called a* fractional *(*g*, *f*, *n*)*-critical graph* if, after deleting any *n* vertices from *G*, the resulting graph still has a fractional (*g*, *f*)-factor. A graph *G* is called a* fractional *(*g*, *f*, *m*)*-deleted graph* if, after deleting any *m* edges, the resulting graph still has a (*g*, *f*)-factor. Fractional deleted graph and fractional critical graph, as extensions of the concept of fractional factor, describe the existence of fractional factor in communication networks when certain channels or certain sites are damaged.

Gao [2] proposed a new concept to deal with the combination situation when some channels and some sites are unavailable in networks. A graph *G* is called a* fractional *(*g*, *f*, *n*, *m*)*-critical deleted graph* if, after deleting any *n* vertices from *G*, the resulting graph is still a fractional (*g*, *f*, *m*)-deleted graph. In particular, the fractional (*g*, *f*, *n*, *m*)-critical deleted graph is just fractional (*g*, *f*, *n*)-critical deleted graph if *m* = 1.

Let

(1) $$\begin{array}{c} \varepsilon \left(S,T\right)=\{\begin{array}{cc} 2, & T\;\;\text{is}\;\;\text{not}\;\;\text{an}\;\;\text{independent}\;\;\text{set}\\ 1, & T\;\;\text{is}\;\;\text{an}\;\;\text{independent}\;\;\text{set},\;\;\text{and}\\ & \;e_{G}\left(T,V\left(G\right)\setminus \left(S\cup T\right)\right)\geq 1\\ 0, & \text{otherwise}. \end{array} \end{array}$$

We heavily depend on the following lemma to prove our main result, which determined a necessary and sufficient condition for a graph to be a fractional (*g*, *f*, *n*)-critical deleted graph.

**Lemma 1 (Gao [2]).**

Let *G* be a graph and let *g*, *f* be two nonnegative integer-valued functions defined on *V*(*G*) satisfying *g*(*x*) ≤ *f*(*x*) for all *x* ∈ *V*(*G*). Let *n* be a nonnegative integer. Then *G* is a fractional (*g*, *f*, *n*)-critical deleted graph if and only if

(2) f(S)−g(T)+dG−S(T) ≥max⁡{f(U):U⊆S,|U|=n}+ε(S,T),

for any disjoint subsets *S* and *T* of *V*(*G*) with |*S* | ≥*n*.

The notion of* toughness* was first introduced by Chvátal in [3] to measure the vulnerable of networks: if *G* is complete graph, *t*(*G*) = *∞*; if *G* is not complete,

(3) $$\begin{array}{c} t\left(G\right)=\min\left\{\frac{\left|S\right|}{\omega \left(G-S\right)}\omega \left(G-S\right)\geq 2\right\}, \end{array}$$

where *ω*(*G* − *S*) is the number of connected components of *G* − *S*.

Liu and Zhang [4] determined a necessary and sufficient condition for a graph to have a fractional (*g*, *f*)-factor. For several characters on fractional (*g*, *f*)-factor one can refer to Liu and Zhang [4, 5] for more details. Liu [6] investigated the necessary and sufficient condition for a graph *G* to be a fractional (*g*, *f*, *n*)-critical graph. For more recent results for fractional deleted graph and fractional critical graph one can refer to [7–12].

Some toughness conditions for a graph to have a fraction factor were given in [13, 14]. Liu et al. [6] studied the relationship between toughness and fractional (*g*, *f*, *n*)-critical graphs and proved that *G* is a fractional (*g*, *f*, *n*)-critical graph if *t*(*G*) ≥ ((*b* ^2^ − 1)(*n* + 1)/*a*) for *a* ≤ *g*(*x*) ≤ *f*(*x*) ≤ *b* with 1 ≤ *a* ≤ *b* and *b* ≥ 2. Zhou et al. [15] studied the toughness condition for fractional (*k*, *m*)-deleted graph. It is determined that *G* is a fractional (*k*, *m*)-deleted graph if *t*(*G*) ≥ *k* + ((2*m* − 1)/*k*). Recently, in [16], Gao et al. derived a new bound for graphs to be fractional (*g*, *f*, *n*)-critical. It is verified that *G* is a fractional (*g*, *f*, *n*)-critical graph if *t*(*G*) ≥ ((*b* ^2^ − 1 + *bn*)/*a*). This inspires us to think about the *t*(*G*) for fractional (*g*, *f*, *n*)-critical deleted graphs. In this paper, we determine that such bound of toughness as above is sufficient for a graph *G* to be a fractional (*g*, *f*, *n*)-critical deleted graph. Our main result to be proved in next section can be stated as follows.

**Theorem 2 .**

Let *G* be a graph and let *g*, *f* be two integer-valued functions defined on *V*(*G*) satisfying *a* ≤ *g*(*x*) ≤ *f*(*x*) ≤ *b* with 1 ≤ *a* ≤ *b* and *b* ≥ 2 for all *x* ∈ *V*(*G*), where *a*, *b* are positive integers. Let *n* be a nonnegative integer. |*V*(*G*)|≥*n* + *b* + 2 if *G* is complete. If *t*(*G*) ≥ ((*b* ^2^ − 1 + *bn*)/*a*), then *G* is a fractional (*g*, *f*, *n*)-critical deleted graph.

Clearly, our result strengthened the previous conclusions, and it is sharp if *a* = *b* and *n* = 0 according to the sharpness example in Liu and Zhang [17]. The proof strategy is similar to the one in Liu and Zhang [17], but we need to cope with the more detailed case now and hence new methods are necessary. Before proving Theorem 2, we would like to show some useful lemmas.

**Lemma 3 (Chvátal [3]).**

If a graph *G* is not complete, then *t*(*G*) ≤ (1/2)*δ*(*G*).

**Lemma 4 (Liu and Zhang [17]).**

Let *G* be a graph and let *H* = *G*[*T*] such that *δ*(*H*) ≥ 1 and 1 ≤ *d*_*G*_(*x*) ≤ *k* − 1 for every *x* ∈ *V*(*H*), where *T*⊆*V*(*G*) and *k* ≥ 2. Let *T*_1_,…, *T*_*k*−1_ be a partition of the vertices of *H* satisfying *d*_*G*_(*x*) = *j* for each *x* ∈ *T*_*j*_, where one allows some *T*_*j*_ to be empty. If each component of *H* has a vertex of degree at most *k* − 2 in *G*, then *H* has a maximal independent set *I* and a covering set *C* = *V*(*H*) − *I* such that

(4) $$\begin{array}{c} \overset{k-1}{\underset{j=1}{\sum }}\left(k-j\right)c_{j}\leq \overset{k-1}{\underset{j=1}{\sum }}\left(k-2\right)\left(k-j\right)i_{j}, \end{array}$$

where *c*_*j*_ = |*C*∩*T*_*j*_| and *i*_*j*_ = |*I*∩*T*_*j*_| for every *j* = 1,…, *k* − 1.

The lemma below can be deduced from Lemma 2.2 in [17].

**Lemma 5 (Liu and Zhang [17]).**

Let *G* be a graph and let *H* = *G*[*T*] such that *d*_*G*_(*x*) = *k* − 1 for every *x* ∈ *V*(*H*) and no component of *H* is isomorphic to *K*_*k*_, where *T*⊆*V*(*G*) and *k* ≥ 2. Then there exist an independent set *I* and the covering set *C* = *V*(*H*) − *I* of *H* satisfying

(5) $$\begin{array}{c} \left|V\left(H\right)\right|\leq \overset{k}{\underset{i=1}{\sum }}\left(k-i+1\right)\left|I^{\left(i\right)}\right|-\frac{\left|I^{\left(1\right)}\right|}{2},\\ \left|C\right|\leq \overset{k}{\underset{i=1}{\sum }}\left(k-i\right)\left|I^{\left(i\right)}\right|-\frac{\left|I^{\left(1\right)}\right|}{2}, \end{array}$$

where *I* ^(*i*)^ = {*x* ∈ *I*, *d*_*H*_(*x*) = *k* − *i*}, 1 ≤ *i* ≤ *k*, and ∑_*i*=1_^*k*^ | *I* ^(*i*)^ | = |*I*|.

## 2. Proof of Theorem 2

If *G* is complete, then *G* is a fractional (*g*, *f*, *n*)-critical deleted graph due to |*V*(*G*)|≥*n* + *b* + 2. In what follows, we assume that *G* is not complete.

Suppose that *G* satisfies the conditions of Theorem 2 but is not a fractional (*g*, *f*, *n*)-critical graph. By Lemma 1 and *ε*(*S*, *T*) ≤ 2, there exist subsets *S* and *T* of *V*(*G*) such that

(6) a|S|+∑x∈TdG−S(x)−b|T| ≤f(S)−g(T)+dG−S(T)≤bn+1.

We choose subsets *S* and *T* such that |*T*| is minimum. Obviously, we deduce *T* ≠ *∅* and *d*_*G*−*S*_(*x*) ≤ *g*(*x*) − 1 ≤ *b* − 1 for any *x* ∈ *T*.

Let *l* be the number of the components of *H*′ = *G*[*T*] which are isomorphic to *K*_*b*_ and let *T*_0_ = {*x* ∈ *V*(*H*′)∣*d*_*G*−*S*_(*x*) = 0}. Let *H* be the subgraph obtained from *H*′ − *T*_0_ by deleting those *l* components isomorphic to *K*_*b*_.

If |*V*(*H*)| = 0, then by virtue of (6) we infer

(7) $$\begin{array}{c} a\left|S\right|\leq b\left|T_{0}\right|+bl+bn+1, \end{array}$$

or

(8) $$\begin{array}{c} \left|S\right|\leq \frac{b\left(\left|T_{0}\right|+l\right)+bn+1}{a}. \end{array}$$

If *ω*(*G* − *S*) = |*T*_0_ | +*l* > 1, then *t*(*G*) ≤ (|*S*|/*ω*(*G* − *S*)) ≤ ((*b*(|*T*_0_ | +*l*) + *bn* + 1)/*a*(|*T*_0_ | +*l*)) < ((*b* + *bn* + 1)/*a*), which contradicts *t*(*G*) ≥ ((*b* ^2^ − 1 + *bn*)/*a*) and *b* ≥ 2. If *ω*(*G* − *S*) = |*T*_0_ | +*l* = 1, then |*T*_0_ | +*l* = 1. Since *d*_*G*−*S*_(*x*)+|*S* | ≥*d*_*G*_(*x*) ≥ *δ*(*G*) ≥ 2*t*(*G*), we have 2*t*(*G*) ≤ *b* − 1 + |*S* | ≤*b* − 1 + ((*b*(*n* + 1) + 1)/*a*), which contradicts *t*(*G*) ≥ ((*b* ^2^ − 1 + *bn*)/*a*).

Now we consider that |*V*(*H*)|>0. Let *H* = *H*_1_ ∪ *H*_2_, where *H*_1_ is the union of components of *H* which satisfies that *d*_*G*−*S*_(*x*) = *b* − 1 for every vertex *x* ∈ *V*(*H*_1_) and *H*_2_ = *H* − *H*_1_. In terms of Lemma 5, *H*_1_ has a maximum independent set *I*_1_ and the covering set *C*_1_ = *V*(*H*_1_) − *I*_1_ such that

(9) $$\begin{array}{c} \left|V\left(H_{1}\right)\right|\leq \overset{b}{\underset{i=1}{\sum }}\left(b-i+1\right)\left|I^{\left(i\right)}\right|-\frac{\left|I^{\left(1\right)}\right|}{2},\\ \left|C_{1}\right|\leq \overset{b}{\underset{i=1}{\sum }}\left(b-i\right)\left|I^{\left(i\right)}\right|-\frac{\left|I^{\left(1\right)}\right|}{2}, \end{array}$$

where *I* ^(*i*)^ = {*x* ∈ *I*_1_, *d*_*H*_1__(*x*) = *b* − *i*}, 1 ≤ *i* ≤ *b*, and ∑_*i*=1_^*b*^ | *I* ^(*i*)^ | = |*I*_1_|. Let *T*_*j*_ = {*x* ∈ *V*(*H*_2_)∣*d*_*G*−*S*_(*x*) = *j*} for 1 ≤ *j* ≤ *b* − 1. Each component of *H*_2_ has a vertex of degree at most *b* − 2 in *G* − *S* by the definitions of *H* and *H*_2_. According to Lemma 4, *H*_2_ has a maximal independent set *I*_2_ and the covering set *C*_2_ = *V*(*H*_2_) − *I*_2_ such that

(10) $$\begin{array}{c} \overset{b-1}{\underset{j=1}{\sum }}\left(b-j\right)c_{j}\leq \overset{b-1}{\underset{j=1}{\sum }}\left(b-2\right)\left(b-j\right)i_{j}, \end{array}$$

where *c*_*j*_ = |*C*_2_∩*T*_*j*_| and *i*_*j*_ = |*I*_2_∩*T*_*j*_| for every *j* = 1,…, *b* − 1. Set *W* = *V*(*G*) − *S* − *T* and *U* = *S* ∪ *C*_1_ ∪ (*N*_*G*_(*I*_1_)∩*W*) ∪ *C*_2_ ∪ (*N*_*G*_(*I*_2_)∩*W*). We infer

(11) $$\begin{array}{c} \left|U\right|\leq \left|S\right|+\left|C_{1}\right|+\overset{b-1}{\underset{j=1}{\sum }}ji_{j}+\overset{b}{\underset{i=1}{\sum }}\left(i-1\right)\left|I^{\left(i\right)}\right|,\\ \omega \left(G-U\right)\geq t_{0}+l+\left|I_{1}\right|+\overset{b-1}{\underset{j=1}{\sum }}i_{j}, \end{array}$$

where *t*_0_ = |*T*_0_|. Let *t*(*G*) = *t*. Then when *ω*(*G* − *U*) > 1, we have

(12) $$\begin{array}{c} \left|U\right|\geq t\omega \left(G-U\right), \end{array}$$

and it also holds when *ω*(*G* − *U*) = 1. In terms of (11) and (12), we get

(13) |S|+|C1| ≥∑j=1b−1(t−j)ij+t(t0+l)+t|I1|−∑i=1b(i−1)|I(i)|.

In view of *b* | *T* | −*d*_*G*−*S*_(*T*) ≥ *a* | *S* | −*bn* − 1, we obtain

(14) bt0+bl+|V(H1)|+∑j=1b−1(b−j)ij+∑j=1b−1(b−j)cj ≥a|S|−bn−1.

Combining with (13), we deduce

(15) bt0+bl+|V(H1)|+∑j=1b−1(b−j)ij  +∑j=1b−1(b−j)cj+a|C1|+bn+1 ≥∑j=1b−1(at−aj)ij+at(t0+l)  +at|I1|−a∑i=1b(i−1)|I(i)|.

Therefore,

(16) |V(H1)|+∑j=1b−1(b−j)cj+a|C1| ≥∑j=1b−1(at−aj−b+j)ij+(at−b)(t0+l)+at|I1|  −a∑i=1b(i−1)|I(i)|−bn−1.

By virtue of (9), we have

(17) |V(H1)|+a|C1| ≤∑i=1b(ab−ai+b−i+1)|I(i)|−(a+1)|I(1)|2.

Using (10), (16), and (17), we get

(18) ∑j=1b−1(b−2)(b−j)ij+∑i=1b(ab−ai+b−i+1)|I(i)| ≥∑j=1b−1(at−aj−b+j)ij+at|I1|+(a+1)|I(1)|2  −a∑i=1b(i−1)|I(i)|+(at−b)(t0+l)−bn−1.

The following proof splits into two cases by the value of *t*_0_ + *l*.

**Case 1 (*t*_0_ + *l* ≥ 1).**

By *at* ≥ *b* ^2^ − 1 + *bn*, we have (*at* − *b*)(*t*_0_ + *l*) − *bn* − 1 ≥ *b* ^2^ − *b* − 2 ≥ 0. Hence, (18) becomes

(19) ∑j=1b−1(b−2)(b−j)ij+∑i=1b(ab−ai+b−i+1)|I(i)| ≥∑j=1b−1(at−aj−b+j)ij+at|I1|  +(a+1)|I(1)|2−a∑i=1b(i−1)|I(i)|.

And then, at least one of the following two cases must hold.

**Subcase 1 (∑_*j*=1_^*b*−1^(*b* − 2)(*b* − *j*)*i*_*j*_ ≥ ∑_*j*=1_^*b*−1^(*at* − *aj* − *b* + *j*)*i*_*j*_).**

There is at least one *j* such that

(20) $$\begin{array}{c} \left(b-2\right)\left(b-j\right)\geq at-aj-b+j, \end{array}$$

which implies

(21) at≤(b−2)(b−j)+aj+b−j=b(b−2)+(a−b+1)j+b.

If *a* = *b*, then *at* ≤ *a*(*a* − 2) + *j* + *a* ≤ *a* ^2^ − 1. By *t*(*G*) ≥ ((*b* ^2^ − 1 + *bn*)/*a*), we get *n* = 0 and ∑_*j*=1_^*b*−2^ *i*_*j*_ = 0, which contradicts the definition of *H*_2_ and the choice of *I*_2_ (see Lemma 4 proof in [17] such that ∑_*j*=1_^*b*−2^ *i*_*j*_ ≠ 0).

If *a* < *b*, then *at* ≤ *b*(*b* − 2)+(*a* − *b* + 1) + *b* = (*b* ^2^ − 1)+(*a* − *b*)+(2 − *b*) < *b* ^2^ − 1, which contradicts *t*(*G*) ≥ ((*b* ^2^ − 1 + *bn*)/*a*).

**Subcase 2 (∑_*i*=1_^*b*^(*ab* − *ai* + *b* − *i* + 1) | *I* ^(*i*)^ | ≥*at* | *I*_1_ | +((*a* + 1) | *I* ^(1)^|/2)−*a*∑_*i*=1_^*b*^(*i* − 1) | *I* ^(*i*)^|).**

If *t*_0_ + *l* ≥ 2 or *b* ≥ 3, then by (*bt* − *a*)(*t*_0_ + *l*) − *bn* − 1 ≥ 1 we have

(22) ∑i=1b(ab−ai+b−i+1)|I(i)| ≥at|I1|+(a+1)|I(1)|2−a∑i=1b(i−1)|I(i)|+1 ≥(b2−1+bn)|I1|+(a+1)|I(1)|2−a∑i=1b(i−1)|I(i)|+1 ≥(b2−1)|I1|+(a+1)|I(1)|2−a∑i=1b(i−1)|I(i)|+1,∑i=2b(ab+b−a−i+2−b2)|I(i)|  +(ab+b−32a−b2+12)|I(1)|≥1.

Let

(23) $$\begin{array}{c} h_{1}\left(b\right)=-b^{2}+\left(a+1\right)b-\frac{3}{2}a+\frac{1}{2}. \end{array}$$

From *b* ≥ *a* and *h*_1_(2) < 0, if *a* = 1, we deduce

(24) $$\begin{array}{c} \max\left\{h_{1}\left(b\right)\right\}=h_{1}\left(a\right)=-\frac{a}{2}+\frac{1}{2}<0. \end{array}$$

Furthermore, *ab* + *b* − *a* − *i* + 2 − *b* ^2^ ≤ −*b* ^2^ + (*a* + 1)*b* − *a* due to *i* ≥ 2. Let

(25) $$\begin{array}{c} h_{2}\left(b\right)=-b^{2}+\left(a+1\right)b-a. \end{array}$$

From *b* ≥ *a*, we infer

(26) $$\begin{array}{c} \max\left\{h_{2}\left(b\right)\right\}=h_{2}\left(a\right)=0. \end{array}$$

This is a contradiction.

If *n* ≥ 1, we obtain

(27) ∑i=1b(ab−ai+b−i+1)|I(i)| ≥(b2−1+bn)|I1|+(a+1)|I(1)|2−a∑i=1b(i−1)|I(i)| ≥(b2−1)|I1|+(a+1)|I(1)|2−a∑i=1b(i−1)|I(i)|+2.

Hence,

(28) ∑i=2b(ab+b−a−i+2−b2)|I(i)| +(ab+b−32a−b2+12)|I(1)|≥2,

a contradiction.

In conclusion, we have *n* = 0, *t*_0_ + *l* = 1, and (*a*, *b*) = (2,2) (if (*a*, *b*) = (1,2), then *h*_1_ < 0 and *h*_2_ < 0, a contradiction). Then the result follows from the main result in [18] which determined that *G* is fractional 2-deleted graph if *t*(*G*) ≥ (3/2).

**Case 2 (*t*_0_ + *l* = 0).**

In this case, (18) becomes

(29) ∑j=1b−1(b−2)(b−j)ij+∑i=1b(ab−ai+b−i+1)|I(i)| ≥∑j=1b−1(at−aj−b+j)ij+at|I1|    +(a+1)|I(1)|2−a∑i=1b(i−1)|I(i)|−bn−1.

*Subcase 1* (|*I*_1_ | = 0). In this subcase, (29) becomes

(30) $$\begin{array}{c} \overset{b-1}{\underset{j=1}{\sum }}\left(\left(b-2\right)\left(b-j\right)-\left(at-aj-b+j\right)\right)i_{j}+bn+1\geq 0. \end{array}$$

Let

(31) hj=(b−2)(b−j)−(at−aj−b+j)=b2+(a−b+1)j−b−at≤b2+(a−b+1)j−b−a·b2−1+bna=(a−b+1)j−b+1−bn.

(i) If *b* ≥ *a* + 1, then

(32) (a−b+1)j−b+1−bn ≤a−2b+2−bn ≤−b+1−bn =−bn−1.

This implies (*a*, *b*) = (1,2), ∑_*j*=2_^*b*−1^ *i*_*j*_ = 0, |*C*_2_ | ≤|*I*_2_|, and |*T* | ≤2 | *I*_2_|. Thus,

(33) $$\begin{array}{c} \left|S\right|\leq \frac{2\left|I_{2}\right|\left(b-1\right)+bn+1}{a}. \end{array}$$

If |*I*_2_ | = 1, then |*S* | ≤((2(*b* − 1) + *bn* + 1)/*a*) = 3 + 2*n* and *δ*(*G*)≤|*S* | +1 ≤ 4 + 2*n*. This contradicts *δ*(*G*) ≥ 2*t*(*G*) ≥ 6 + 4*n*. Hence, |*I*_2_ | ≥2.

Let *Z* = {*x*∣*x* ∈ *C*_2_, *d*_*G*−*S*_(*x*) = 1} and *z* = |*Z*|. Thus, 0 ≤ *z* ≤ |*I*_2_| and *N*_*G*−*S*_(*v*) ∈ *I*_2_ if *v* ∈ *Z*. We obtain

(34) $$\begin{array}{c} \left|S\right|\leq \frac{\left(\left|I_{2}\right|+z\right)\left(b-1\right)+\left(\left|C_{2}\right|-z\right)\left(b-2\right)+bn+1}{a}. \end{array}$$

Letting *Z*′ = {*x*∣*x* ∈ *N*_*G*_(*I*_2_)∩*W*, *d*_*G*−*S*_(*x*) = 1}, we infer

(35) b2−1+bna ≤t(G)≤|U−Z∪Z′|ω(G−(U−Z∪Z′)) ≤((|I2|+z)(b−1)+(|C2|−z)(b−2)+bn+1a    +(|C2|−z)+(|I2|−|C2|))×|I2|−1.

By (*a*, *b*) = (1,2) and |*C*_2_ | ≤|*I*_2_|, we get 2*n*(1 − (1/|*I*_2_|)) ≤ (1/|*I*_2_|) − 1. By |*I*_2_ | ≥2, we derive the contradiction.

(ii) If *a* = *b*, then max⁡{*h*_*j*_} = *h*_*b*−1_ = −*bn* and the second largest value of *h*_*j*_ is *h*_*b*−2_ = −*bn* − 1. In terms of the analysis of Lemma 4 in [17]: for each connected component of *H*_2_, choose a vertex with the smallest degree and add it to *I*_2_. Hence, by the definition of *H*_2_, we confirm that *H*_2_ is connected; each vertex in *I*_2_ has degree *b* − 1 in *G* − *S* except that one vertex has degree *b* − 2 in *G* − *S*. This fact implies

(36) $$\begin{array}{c} \left|C_{2}\right|\leq \left(b-2\right)+\left(\left|I_{2}\right|-1\right)\left(b-1-1\right)=\left|I_{2}\right|\left(b-2\right),\\ \left|T\right|\leq \left|I_{2}\right|\left(b-1\right),\\ \left|S\right|\leq \frac{\left|T\right|+1+bn}{a}\leq \left|I_{2}\right|+\frac{1-\left|I_{2}\right|}{b}+n. \end{array}$$

If |*I*_2_ | = 1, then |*S* | ≤1 + *n* and *δ*(*G*)≤|*S* | +(*b* − 1) ≤ *b* + *n*, which contradicts *δ*(*G*) ≥ 2*t*(*G*) > *b* + *n*. Hence, |*I*_2_ | ≥2 and

(37) b−1b+n≤t(G)≤|U|ω(G−U)≤((1−|I2|)/b)+|I2|+|I2|(b−2)+n|I2|=(b−1−1b)+1b|I2|+n|I2|.

This reveals *n*(1 − (1/|*I*_2_|))≤((1/*b*|*I*_2_|) − 1), which contradicts *b* ≥ 2 and |*I*_2_ | ≥2.

*Subcase 2* (|*I*_2_ | = 0). In this subcase, (29) becomes

(38) ∑i=1b(ab−ai+b−i+1)|I(i)|−at|I1|−(a+1)|I(1)|2  +a∑i=1b(i−1)|I(i)|+bn+1≥0.

This implies

(39) ∑i=2b(ab+b−a−i+2−b2)|I(i)|  +(ab+b−32a−b2+12)|I(1)|+1≥0.

Then, by *h*_1_ ≤ −1/2, we get ∑_*i*=4_^*b*^ | *I* ^(*i*)^ | = 0, |*I* ^(3)^ | ≤1, and |*I* ^(1)^ | ≤2. Now, we consider the following three subcases.

*Subcase 2* *.1* (|*I* ^(1)^ | = 1). In this subcase, we have ∑_*i*=3_^*b*^ | *I* ^(*i*)^ | = 0. By analyzing the proof of Lemma 2.2 in [17]: “for each vertex *x* ∈ *I*_*n*_ and *d*_*H*_*n*__(*x*) = *k* − 1, there exists a vertex *y* ∈ *I*_*n*_ such that *N*_*H*_*n*__(*x*)∩*N*_*H*_*n*__(*y*) ≠ *∅*,” we obtain |*I*_1_ | ≥2,

(40) |T|≤(b−1)+(|I1|−1)(b−1)=|I1|(b−1),|S|≤|T|+1+bna≤|I1|(b−1)+1+bna,|U|≤|S|+|C1|+∑i=1b(i−1)|I(i)|≤|I1|(b−1)+1+bna+|I1|(b−1)−|I1|+(|I1|−1)=|I1|(b−1)+1+bna+|I1|(b−1)−1.

Thus,

(41) b2−1+bna ≤t(G)≤|U|ω(G−U) ≤((|I1|(b−1)+1+bn)/a)+|I1|(b−1)−1|I1|.

This implies *bn*(1 − (1/|*I*_1_|))≤(*b* − *a*)(1 − *b*) + ((1 − *a*)/|*I*_1_|), a contradiction.

*Subcase 2.2* (|*I* ^(1)^ | = 2). In this subcase, ∑_*i*=3_^*b*^ | *I* ^(*i*)^ | = 0. We can get a contradiction via a similar discussion as in Subcase 2.1.

*Subcase 2.3*(|*I* ^(1)^| = 0). In this subcase, we have ∑_*i*=4_^*b*^ | *I* ^(*i*)^ | = 0 and |*I* ^(3)^ | ≤1. If |*I*_1_ | = 1, then |*S* | ≤(((*b* − 1) + *bn* + 1)/*a*). Thus, we infer

(42) (b−1)+bn+1a+b−1 ≥b−1+|S|≥δ(G)≥2t(G) ≥2(b2−1+bn)a,

a contradiction. Hence, |*I*_1_ | ≥2. Let *Y* = *N*_*G*_(*I*_1_)∩*W*.

If there is a vertex *y* ∈ *Y* such that *y* only adjacent to one vertex in *I*_1_. Reset

(43) $$\begin{array}{c} U=S\cup C_{1}\cup \left(N_{G}\left(I_{1}\right)\cap \left(W-\left\{y\right\}\right)\right). \end{array}$$

Then, we have

(44) |U|≤|S|+|I1|(b−1)−1≤|I1|(b−1)+1+bna+|I1|(b−1)−1.

By |*I*_1_ | ≥2, we obtain

(45) b2−1+bna ≤t(G)≤|U|ω(G−U) ≤((|I1|(b−1)+1+bn)/a)+|I1|(b−1)−1|I1|.

This implies *bn*(1 − (1/|*I*_1_|))≤(*b* − *a*)(1 − *b*) + ((1 − *a*)/|*I*_1_|), a contradiction.

If each vertex in *Y* is adjacent to at least two vertices in *I*_1_, we get

(46) |U|≤|S|+|I1|(b−2)+|I1|2≤|I1|(b−1)+1+bna+|I1|(b−2)+|I1|2,

where *U* = *S* ∪ *C*_1_ ∪ (*N*_*G*_(*I*_1_)∩*W*). Due to |*I*_1_ | ≥2, we deduce

(47) b2−1+bna ≤t(G)≤|U|ω(G−U) ≤((|I1|(b−1)+1+bn)/a)+|I1|(b−2)+(|I1|/2)|I1|.

That is to say, *bn*(1 − (1/|*I*_1_|))≤(*b* − *a*)(1 − *b*)+((1/|*I*_1_|) − (*a*/2)), which contradicts *a* ≥ 1 and |*I*_1_ | ≥2.

*Subcase 3* (|*I*_1_ | ≠0 and |*I*_2_ | ≠0). From what we have discussed in Subcase 1, we get ∑_*j*=1_^*b*−1^(*b* − 2)(*b* − *j*)*i*_*j*_ ≤ ∑_*j*=1_^*b*−1^(*at* − *aj* − *b* + *j*)*i*_*j*_ + *bn* + 1. Then, we deduce

(48) ∑i=1b(ab−ai+b−i+1)|I(i)| ≥at|I1|+(a+1)|I(1)|2−a∑i=1b(i−1)|I(i)|.

This implies

(49) ∑i=2b(ab+b−a−i+2−b2)|I(i)|  +(ab+b−32a−b2+12)|I(1)|≥0.

Thus, we have ∑_*i*=4_^*b*^ | *I* ^(*i*)^ | = 0, |*I* ^(3)^ | ≤1, |*I* ^(1)^ | ≤2, and *n* = 0, by what we have discussed in Subcase 2. It is enough to discuss the situation of |*I* ^(1)^ | = 0; other two cases for |*I* ^(1)^ | = 1 and |*I* ^(1)^ | = 2 can be considered in a similar way.

Under the condition of |*I* ^(1)^ | = 0, we get ∑_*i*=4_^*b*^ | *I* ^(*i*)^ | = 0, |*I* ^(3)^ | ≤1,

(50) $$\begin{array}{c} \left|T\right|\leq \left|I_{1}\right|\left(b-1\right)+\left|I_{2}\right|\left(b-1\right)=\left(b-1\right)\left(\left|I_{1}\right|+\left|I_{2}\right|\right),\\ \left|S\right|\leq \frac{\left|T\right|+1}{a}\leq \frac{\left(\left|I_{1}\right|+\left|I_{2}\right|\right)\left(b-1\right)+1}{a}. \end{array}$$

Since |*I*_1_ | +|*I*_2_ | ≥2, we get

(51) b2−1a≤t(G)≤|U|ω(G−U)≤|S|+|I2|(b−2)+|I1|(b−1)|I1|+|I2|.

Hence,

(52) (b2−1)(|I1|+|I2|) ≤(|I1|+|I2|)(b−1)+1+(ab−2a)(|I1|+|I2|)  +|I1|a.

This implies (*b* − *a*)(*b* − 1)(|*I*_1_ | +|*I*_2_|) ≤ 1 − *a* | *I*_2_|, a contradiction.

We complete the proof of the theorem.
